# Supplementary material for: The Systems Biology Research Tool: evolvable open-source software
Source: BMC Syst Biol. 2008 Jun 29;2:55. doi: 10.1186/1752-0509-2-55 (PMC2446383; doi:10.1186/1752-0509-2-55)
Supplement: Additional file 1 — SBRT Archive. An archive of the current version of the Systems Biology Research Tool. [file 1752-0509-2-55-S1.zip › sbrt-1.4.0/doc/index.html]

The Systems Biology Research Tool


# The Systems Biology Research Tool Documentation

|  |  |
| --- | --- |
| Documents | Brief Descriptions |
| User's Guide | Describes the way in which the Systems Biology Research Tool can be used as an application. |
| Developer's Guide | Describes the way in which the Systems Biology Research Tool can be used by software developers. |
|  |  |
